# Supplementary material for: Multiparametric Comparison of Two TTA-Based Surgical Techniques in Dogs with Cranial Cruciate Ligament Tears
Source: Animals (Basel). 2023 Nov 9;13(22):3453. doi: 10.3390/ani13223453 (PMC10668821; doi:10.3390/ani13223453)
Supplement: Supplementary file 1 [file animals-13-03453-s001.zip › animals-2675425-supplementary.pdf]

Table S1. Distribution of dogs by gender.

Sex	Group 1	Group 2	Total
Male non-spayed	6	8	14
Male spayed	1	0	1
Female non-spayed	6	2	8
Female spayed	2	5	7

Table S2. Distribution of dogs by breed.

Breed	Group 1	Group 2	Total
American Staffordshire Terrier	1		1
Boxer	1	3	4
Carea Leones		1	1
Dalmatian		1	1
Doberman		1	1
Golden Retriever	2		2
German Shepherd		1	1
Husky	1		1
Labrador		1	1
Spanish Mastin	1		1
Mixed	5	2	7
Pachón Navarro	1		1
Spanish Water Dog	2	1	3
Pitbull Terrier		1	1
English Setter	1		1
Irish Setter		1	1
West Highland Terrier		2	2

Table S3. Distribution of dogs by side of disease..

Knee	Group 1	Group 2	Total
Right	6	8	14
Left	9	7	16

Table S4. Distribution of dogs based on physical examination.

	Group 1	Group 2	Total	Percentage (%)
PAIN				
yes	12	11	23	76,7
no	3	4	7	23,3
INFLAMMATION				
yes	13	14	27	90
no	2	1	3	10
JOINT LEAK				
yes	13	12	25	83,3
no	2	3	5	16,7
FINOCHIETTO				
yes	5	6	11	36,67
no	9	10	19	63,33
Drawer test				
absent	3	2	5	16,67
present	8	8	16	53,33
clear	4	5	9	30

Table S5. Changes in the affected limb while standing, before de surgery and 3 months after surgery. (0) Dog stands normal (1) Dog shuffles weight to one side (2) Dog only supports his limb with his fingers (3) Dog doesn't support the hind.

Score before the surgery	Group 1	Group 2	Total
0	0	0	0
1	8	6	14
2	5	7	12
3	2	2	4
Mean score	1,6	1,73	
Score 3 months after surgery	Group 1	Group 2	Total
0	10	12	22
1	5	3	8
2	0	0	0
3	0	0	0
Mean score	0,3	0,2	

Table S6. Changes of posture while getting up, before and 3 months after surgery. (0) Dog gets up normally (1) Dog changes posture while getting up (2) Dog has severe difficulty to get up (3) Dog doesn't get up.

Score before surgery	Group 1	Group 2	Total
0	3	2	5
1	9	8	17
2	3	5	8
3	0	0	0
Mean sore	1	1	
Score 3 months after surgery	Group 1	Group 2	Total
0	8	8	16
1	7	6	13
2	0	1	1
3	0	0	0
Mean score	0,47	0,53	

Table S7. Lameness before surgery and 3 months after surgery. (0) No lameness, (1) Limps at the begging but stops during 10 minutes of walk (2) Limps a the beginning but stops after 10 minutes of walk (3) The limp never stopes.

Score before surgery	Group 1	Group 2	Total
0	0	0	0
1	6	6	6
2	1	2	2
3	8	7	22
Mean score	2,13	2,07	
Score 3 months after surgery	Group 1	Group 2	Total
0	8	7	18
1	5	6	8
2	0	0	0
3	2	2	4
Mean score	0,73	0,8	

Table S8. Lameness after 10 minutes of walk, before and 3 months after surgery. (0) No lameness (1) minor limp (2) severe limp (3) doesn't support the affected limb.

Score before surgery	Group 1	Group 2	Total
0	1	2	2
1	6	6	8
2	5	4	9
3	3	3	12
Mean score	1,67	1,53	
Score 3 months after surgery	Group 1	Group 2	Total
0	12	10	22
1	2	5	7
2	0	0	0
3	1	0	1
Mean score	0,33	0,33	

Table S9. Resistance to walk, before and 3 months after surgery. (0) Can take long walks (1) During long walks must stop often (2) can only tolerate short walks (less than 10 minutes), (3) Doesn't want to walk.

Score before surgery	Group 1	Group 2	Total
0	6	7	8
1	6	5	9
2	2	2	8
3	1	1	5
Mean score	0,87	0,8	
Score 3 months after surgery	Group 1	Group 2	Total
0	12	14	26
1	1	0	1
2	1	1	2
3	1	0	1
Mean score	0,4	0,13	

Table S10. Resistance to run and play, before surgery and the 3 months after. (0) Runs and plays without difficulty (1) Runs and plays with some difficulty (2) Runs and plays with severe difficulty (3) doesn't run or play.

Score before surgery	Group 1	Group 2	Total
0	0	1	1
1	2	7	9
2	11	4	15
3	2	3	5
Mean score	1,67	1,6	
Score 3 months after surgery	Group 1	Group 2	Total
0	11	10	21
1	3	5	8
2	1	0	1
3	0	0	0
Mean score	0,33	0,33	

Table S11. Resistance to climb stairs before surgery and the 3 months after. (0) Climbs stairs without difficulty (1) Climbs long number of stairs (>16) with some difficulty (2) Difficulty to climb short number of stairs (<3), (3) doesn't climb stairs.

Score before surgery	Group 1	Group 2	Total
0	3	3	6
1	8	6	16
2	2	3	4
3	2	3	4
Mean score	1,2	1,4	
Score 3 months after surgery	Group 1	Group 2	Total
0	11	13	24
1	4	2	6
2	0	0	0
3	0	0	0
Mean score	0,27	0,13	

Table S12. Limitation to take small jumps (to the car o sofa), before surgery and 3 months after. (0) Can jump without a problem (1) Can jump with difficulty (2) can´t jump.

Score before surgery	Group 1	Group 2	Total
0	3	4	7
1	8	6	15
2	4	5	9
Mean score	1,07	1,07	

Score 3 months after surgery	Group 1	Group 2	Total
0	15	13	28
1	0	2	2
2	0	0	0
Mean score	0	0,13	

Table S13. Manual articular mobility of the stifle, before and 3 months after surgery. (0) no pain, no crepitation, (1) Slight pain on the last degrees of extension and flexion of the stifle (2) Pain and crepitation during the entire movement (3) Can't flex or extend the stifle due to the severe pain.

Score before surgery	Group 1	Group 2	Total
0	1	1	2
1	9	8	18
2	4	5	8
3	1	1	2
Mean score	1,33	1,4	
Score 3 months after surgery	Group 1	Group 2	Total
0	12	14	26
1	2	0	2
2	1	1	2
3	0	0	0
Mean score	0,27	0,13	

Table S14. Limitation of the articular flexion movement in degrees, before and 3 months after surgery. (0) total flexion 40°-50°, (1) minor limitation to flex <70°, (2) sever limitation to flex >70°.

Score before surgery	Group 1	Group 2	Total
0	3	4	7
1	11	10	16
2	1	1	7
Mean score	0,87	0,73	
Score 3 months after surgery	Group 1	Group 2	Total
0	13	13	26
1	2	2	4
2	0	0	0
Mean score	0,13	0,13	

Table S15. Limitation of the articular extension movement in degrees, before and 3 months after surgery. (0) total extension 160°-1700°, (1) minor limitation to extend <150°, (2) sever limitation to extend >150°.

Score before surgery	Group 1	Group 2	Total
0	3	4	4
1	11	10	21
2	1	1	5
Mean score	0,87	0,8	
Score 3 months after surgery	Group 1	Group 2	Total
0	14	14	28
1	1	1	2
2	0	0	0
Mean score	0,07	0,07	

Table S16. Muscular atrophy before and 3 months after surgery. (0) no signs of muscular atrophy, (1) minor muscular atrophy, (2) severe muscular atrophy.

Score before surgery	Group 1	Group 2	Total
0	7	7	13
1	7	8	15
2	1	0	2
Mean score	0,6	0,53	
Score 3 months after surgery	Group 1	Group 2	Total
0	11	13	24
1	3	2	5
2	1	0	1
Mean score	0,33	0,13	

Table S17. Average OA score, before, one month and 3 months after surgery. (0-2) no signs of OA, (3-8) minor arthrosis, (9-18) mild arthrosis, (>18) severe arthrosis.

	G	1			G	2	
Dog	Surgery day	1 month	3 months	Dog	Surgery day	1 month	3 months
1	4	5	6	16	34	34	34
2	17	17	17	17	4	4	4
3	34	34	34	18	21	21	21
4	9	9	9	19	2	2	2
5	1	1	1	20	18	18	18
6	7	7	7	21	9	9	9
7	9	9	9	22	6	7	7
8	3	3	3	23	2	2	2
9	9	9	9	24	9	9	9
10	3	3	3	25	2	2	2
11	10	10	10	26	10	10	10
12	5	5	5	27	7	7	7
13	13	13	13	28	4	4	4
14	9	9	9	29	6	6	6
15	11	11	11	30	4	4	4

Table S18. Osteointegration of the implant in the first and third month after surgery.

Osteointegration 1 month	Group 1	Group 2	Total
Good	13	13	26
mild/bad	2	2	4
Osteointegration 3 month			
good	14	14	28
Mild/bad	1	1	2

Table S19. Minor complications post-surgery.

	Group 1	Group 2	Total
Superficial wound	1	0	1
Wound dehiscence	0	0	0
Seroma	0	0	0
Others	0	0	0

Table S20. Major complications post-surgery

	Group 1	Group 2	Total
Implant fails	1	0	1
Osteomyelitis	0	0	0
Avulsion of Tibial crest	0	1	1
Others	0	0	0

Table S21. Surgery duration (minutes).

Dog	Group 1	Dog	Group 2
1	41	16	40
2	40	17	40
3	45	18	43
4	42	19	47
5	44	20	48
6	39	21	39
7	40	22	46
8	52	23	49
9	53	24	47
10	42	25	42
11	43	26	45
12	42	27	42
13	43	28	41
14	43	29	45
15	40	30	40
Total	649	Total	654
Average	43,26	Average	43,60
